# Color and Nutritional Analysis of Ten Different Purple Sweet Potato Varieties Cultivated in China via Principal Component Analysis and Cluster Analysis

**DOI:** 10.3390/foods13060904

**Published:** 2024-03-16

**Authors:** Meng Xu, Jia Li, Jinjing Yin, Muci Wu, Wangting Zhou, Xinsun Yang, Rui Zhang, Jingren He

**Affiliations:** 1 National R&D Center for Se-Rich Agricultural Products Processing, Hubei Engineering Research Center for Deep Processing of Green Se-Rich Agricultural Products, School of Modern Industry for Selenium Science and Engineering, Wuhan Polytechnic University, Wuhan 430023, China; xumeng9902@163.com (M.X.); lijia331@163.com (J.L.); oliviayin1007@163.com (J.Y.); muciwu@whpu.edu.cn (M.W.); zhouwangting01@whpu.edu.cn (W.Z.); 2 Institute of Food Crops, Hubei Academy of Agricultural Sciences, Wuhan 430064, China; yangxins013@163.com; 3 Key Laboratory for Deep Processing of Major Grain and Oil, Ministry of Education, Hubei Key Laboratory for Processing and Transformation of Agricultural Products, Wuhan Polytechnic University, Wuhan 430023, China

**Keywords:** purple sweet potatoes, nutritional components, anthocyanin composition, principal component analysis, cluster analysis

## Abstract

Purple sweet potato (PSP) has abundant nutritional compounds, which are valuable constituents of the human diet, but its development and utilization are still in the primary processing phase. This study examined the differences in nutritional characteristics of 10 PSP varieties. A variety of nutritional components were evaluated and comprehensively compared using principal component analysis (PCA) and cluster analysis (CA). The PSP had 60.9–70.1% moisture. The dried PSP had abundant starch (43.9~67.2%) and dietary fiber (9.40~16.5%), moderate levels of protein (3.19~8.75%) and reducing sugar (1.44~4.01%), and low amounts of crude fat (0.51~1.01%). The anthocyanin profile varied significantly between the different varieties. A correlation analysis showed that a higher content of anthocyanins resulted in a darker color. The PCA and CA suggested that varieties XS, ZL, and JS18 are desirable for developing the diabetic patient’s diet. JS1 had the highest anthocyanin, protein, and dietary fiber contents and the lowest starch, implying that it could be used as a source of natural colorants or functional foods. Varieties FX, GS, ES13, and EN are suitable for producing various starch-based food products, such as noodles, cookies, and pastries. This study provides a reference for the practical use and rational processing of PSP resources.

## 1. Introduction

Purple sweet potato (PSP) (*Ipomoea batatas* L.), a member of the sweet potato family, exhibits an intense purple color in the root and skins owing to the accumulation of anthocyanins [1]. As one of the most promising economic crops, PSP contains abundant amounts of nutrients. Owing to its high nutritional content and ability to promote health, a number of new varieties have been developed to meet the demands of consumers for healthier foods [2]. Currently, varieties such as Jishu No. 18, Guangshu No. 1, and Eshu No. 13 have been widely cultivated in Shandong, Guangxi, and Hubei Provinces in China. An increasing number of researchers have chosen PSP as the raw material for developing new products. 

In the current market, PSPs have been applied to various fields for both fresh consumption and primary food processing. For example, starch is one of the primary components in PSPs, which results in their use to produce various starch-based food products, such as noodles [3], cookies [4], and pastries [5]. Starch consists of digestible and indigestible portions. The latter is also called resistant starch because it resists digestion in the small intestine and is completely or partially fermented in the colon. The health benefits related to the consumption of resistant starch include the prevention of colon cancer, effects on hypoglycemia, reduction in gallstone formation, and the increased absorption of minerals [6,7]. In addition, PSPs have a high content of anthocyanins. As is well known, anthocyanins are natural colorants with multiple bioactive properties, including their use as antioxidants [8] and scavengers of free radicals [9], their ability to protect and prevent liver injury [10], and other physiological functions. Hu et al. [7] compared and characterized the antioxidant activities of the anthocyanins in 30 varieties of PSPs. They reported that four peonidin-based anthocyanins and three cyanidin-based anthocyanins in PSP contribute significantly to its antioxidant activity. Afiati et al. [11] reported that the addition of 2% of anthocyanin extracts of PSP to yogurt could improve its functional characteristics. Zhu et al. [12] also found that the addition of PSP flour to Chinese steamed bread (CSB) could increase its antioxidant activities and reduce the glycemic response. Moreover, Guclu et al. [13] investigated the effects of different processing methods on differently colored sweet potatoes. They found that the treated PSP had the highest total phenolic content and twice the antioxidant capacity of orange or white sweet potatoes.

However, most studies have focused on exploring or comparing the nutritional composition of single or multiple varieties without establishing a relevant and differentiated evaluation system. This lack of a comprehensive evaluation system hinders the rational development and utilization of PSP resources. Therefore, implementing a systematic evaluation of the differences in PSP varieties can provide a solid foundation for future research. To achieve this aim, 10 varieties of PSPs from different environments were selected to compare their differences in the aspects of color parameters and nutritional components, including moisture, protein, crude fat, starch, reducing sugar, total dietary fiber, anthocyanins, and selenium, using principal component analysis (PCA) and cluster analysis (CA). The varieties with the highest contents of anthocyanins were further analyzed for their structural composition using HPLC-DAD/ESI-MS^2^. This study aimed to comprehensively understand the nutritional and color characteristics among the different varieties and provide theoretical guidance for the practical use of PSP resources. 

## 2. Materials and Methods

### 2.1. Materials

Ten types of PSP, including the varieties Eshu No. 12 (ES12) and Eshu No. 13 (ES13) (Wuhan, Hubei), Zi luolan (ZL) (Nanyang, Henan), Xushu No. 1 (XS) (Xuzhou, Jiangsu), Fu xi (FX) (Ganzhou, Jiangsu), Guangshu No. 1 (GS) (Shangrao, Jiangxi), Jishu No. 18 (JS18), Jishu No. 1 (JS1), Jishu No. 2 (JS2) (Linyi, Shangdong), and Enshi (EN) (Enshi, Hubei) were collected from five different provinces, which were cultivated in the middle and lower reaches of Yangtze river and central China. They were provided by the Institute of Food Crops, Hubei Academy of Agricultural Sciences (Wuhan, China). Except for the measurements of color and moisture, all the samples were pulverized by a powder tester before analysis, followed by passage through a 100-mesh sieve and storage in a dryer. Each sample was tested in triplicate.

### 2.2. Reagents 

Hydrochloric acid (HCl) (GR, 36–38%), ethanol (AR, 99.8%), potassium chloride (KCl) (AR, 99.5%), acetic acid (AR, 99.5%), sodium acetate (AR, 99.0%), cupric tartrate (AR, 99%), petroleum ether (AR, 30–60 °C), iodine (0.1 N), sodium hydroxide (NaOH) (AR, 96%), zinc acetate (AR, 99%), potassium cyanide (AR, 30–60 °C), glucose (AR, 99.9%), ammonia sulfate (AR, 99%), acetone (AR, >99.5%), and nitric acid (GR, 65–68%) were obtained from Sinopharm Chemical Reagent Co., Ltd. (Shanghai, China). Methanol (HPLC, 99.8%), acetonitrile (HPLC, 99.8%), and formic acid (HPLC, 99%) were purchased from Shanghai Xingke High Purity Solvent Co., Ltd. (Shanghai, China). α-Amylase (500 KU), protease (500 KU), and glucoamylase (500 KU) were provided by Beijing Huamaike Biotechnology Co., Ltd. (Beijing, China), Shanghai Macklin Biochemical Technology Co., Ltd. (Shanghai, China) and Shanghai Aladdin Bio-Chem Technology Co., Ltd. (Shanghai, China), respectively.

### 2.3. Physical Parameters of the Fresh Samples

#### 2.3.1. Color Analysis

The first step was to clean the surfaces of the samples of different varieties of PSPs by washing off the sediment. The edible parts were then cut into pieces of comparable sizes. These pieces were photographed on a D5300 Nikon digital camera (Nikon, Tokyo, Japan). The colors of these pieces were then measured in the CIELAB uniform space using an UltraScan VIS HunterLab colorimeter (Reston, VA, USA) to record “L*” (lightness), “a*” (−a greenness and +a redness) and “b*” (−b blueness and +b yellowness) values.

#### 2.3.2. Moisture Content

The moisture content was measured as described in the National Food Safety Standards of China (GB 5009.3-2010) [14]. Briefly, 2 g (accurate to ±0.0001 g) of the sliced PSP was weighed in the aluminum case and dried in a 105 °C oven for 4 h. The samples were then removed from the dryer, cooled to room temperature, and weighed. The drying process was repeated until the weights differed by ≤2 mg, which was considered to indicate a constant weight. The moisture content was calculated using Equation (1):

Moisture content (%) = 100 × (m_1_ − m_2_)/(m_1_ − m_3_) (1)

where *m*_1_ is the total mass of the aluminum case and sample (g), *m*_2_ is the total mass of the aluminum case and sample after drying (g), and *m*_3_ is the mass of the aluminum case (g).

### 2.4. Chemical Composition of the Dried Samples

The dried samples used for chemical composition analysis were obtained through freeze drying (Alpha 2–4 LSC plus, Martin Christ Gefriertrocknungsanlagen, Burladignen, Germany) at −80 °C for 72 h with a chamber pressure of 0.05 mbar.

#### 2.4.1. Determination of the Starch Content

The starch content was determined as described in the National Food Safety Standards in China (GB 5009.9-2016) [15]. First, 2 g (accurate to 0.0001 g) of the sample was mixed with 50 mL of petroleum ether and 150 mL of 85% (*v*/*v*) ethanol to remove the fat and soluble sugars. The residue was then washed in a beaker with 50 mL of distilled water. The mixture was boiled for 15 min, cooled to 60 °C, and incubated at room temperature for 1 h with the addition of 20 mL of α-amylase (5 g/L). The mixture was then boiled to inhibit the enzymes and cooled to room temperature. The mixture was transferred to a 250 mL bottle to determine its volume.

Secondly, 5 mL of 6 mol/L HCl was added to 50 mL of filtrate taken from the treated supernatant and refluxed for 1 h in a boiling water bath. The solution was then cooled to room temperature and adjusted to neutral pH with 20% NaOH with two drops of methyl red indicator solution. It was then diluted to 100 mL with distilled water.

Finally, alkaline copper tartrate A (5 mL), alkaline copper tartrate B (5 mL), and distilled water (10 mL) were added to titrate the sample until the blue color just faded to the endpoint on an electric furnace. Two glass beads were added to prevent the solution from boiling. The volume of sample consumption was recorded. The starch content was determined using Equation (2):

Starch content (%) = 100 × (A_1_ − A_2_) × 0.9/[m_1_ × (50/250) × (V_1_/100) × 1000] (2)

where *A*_1_ is the content of reducing sugar in the sample (mg), *A*_2_ is the content of reducing sugar in the blank (mg), *V*_1_ is the volume of the sample titrated (mL), *m*_1_ is the quality of the sample (g), and 0.9 is the conversion coefficient used to convert the starch to reducing sugar.

#### 2.4.2. Determination of the Contents of Reducing Sugar

The contents of reducing sugar were determined as described in the National Food Safety Standards in China (GB 5009.7-2016) [16]. Briefly, 15 g of sample was added to 200 mL of distilled water and heated at 45 °C in a water bath for 1 h. Deionized water was added to bring the volume to 250 mL after the mixture had been cooled to room temperature. A volume of 200 mL of the liquid supernatant was removed from a volumetric flask with 5 mL of zinc acetate and 5 mL of potassium ferrocyanide. A sufficient quantity of deionized water was added to bring the volume up to 250 mL. Next, the suspension was poured through filter paper in a funnel and connected to a suction flask and vacuum system. After suction filtration, the filter liquor was used for titration, as described in Section 2.4.1. The reduced sugar content was calculated using Equation (3):

Reducing sugar content (g/100 g) = 100 × m_1_/[m × F × (V/250)] (3)

where *m*_1_ is the mass of the reducing sugar contained in the alkaline copper tartrate solution (mg), *m* is the mass of the sample (g), *F* is a coefficient equal to 1 in this experiment, and *V* is the average sample volume that was consumed during the measurement (mL).

#### 2.4.3. Determination of the Content of Total Dietary Fiber 

The content of total dietary fiber in the sample was determined as described in the National Food Safety Standards in China (GB 5009.88-2014) [17]. Briefly, 40 mL of MES-TRIS (0.05 mol/L) was added to 1 g of sample (accurate to ±0.0001 g), and duplicate samples of each group were prepared. The difference between the two samples is ≤0.005 g. The mixture was predigested by 50 L of thermostable amylase (1000 U/mL) at 95 °C for 30 min, 10 L of protease (300–400 U/mL) at 60 °C for 30 min and 100 L of glucoamylase (2000–3300 U/mL) at 60 °C for 30 min. 

After the reaction, each enzymatic hydrolysate was precipitated for 1 h at room temperature by the addition of 200 mL of 95% (*v*/*v*) ethanol. The mixture was poured into a diatomite filter and washed with sequential treatments of 30 mL of 78% (*v*/*v*) ethanol, 30 mL of 95% (*v*/*v*) ethanol, and 30 mL of acetone. The diatomite filter that was covered with precipitate was dried at 105 °C for 12 h. The heated samples were used to measure the content of protein and ash after they had cooled to room temperature. The total dietary fiber content was calculated as described in Equation (4):

Total dietary fiber content (g/100 g) = (m_R_ − m_P_ − m_A_ − m_B_)/(m × f) (4)

where *m_R_* is the average quality of residue in the sample (g), *m_P_* is the quality of protein in the sample (g), *m_A_* is the quality of ash in the sample (g), *m_B_* is the quality of the reagent blank (g); *m* is the average quality of dual samples (g), and *f* is a correction factor in this experiment.

#### 2.4.4. Determination of the Content of Protein

The protein content of PSP was quantified using an automated Udk159 Kjeldahl system (VELP Scientifica Srl, Usmate Velate, Italy). In this study, 0.5 g of the samples were predigested at 40 °C for 10 min, followed by treatment at 270 °C for 1 h and 480 °C for 2 h. The generated nitrogen was received by 33% of the NaOH solution and 4% of the boric acid indicator. The solution was titrated with 0.1 mol/L HCl. The protein content was determined using Equation (5):

(5) $$\mathrm{Protein}\;\mathrm{content}\;(\%)=\frac{\left(v_{1}-v_{2}\right)\times c\times 0.0140}{m\;\times \;v_{3}/100}\times F\times 100$$

where *v*_1_ is the volume of HCl standard titration solution consumed by the test solution (mL), *v*_2_ is the volume of HCl standard titrant consumed by the reagent blank (mL), *c* is the concentration of HCl standard titration solution (mol/L), 0.0140 is the mass of nitrogen equivalent to 1.0 mol/L HCl standard titration standard solution (g), *m* is the mass of the sample (g), *v*_3_ is the volume of aspiration digest (mL), and *F* is the coefficient of nitrogen conversion to protein (6.25).

#### 2.4.5. Determination of the Content of Crude Fat

The content of crude fat in the sample was determined as described in the National Food Safety Standards in China (GB 5009.6-2016) [18]. Generally, 3 g of dried sample (accurate to ±0.0001 g) was covered with filter paper in the extraction cylinder of the Soxhlet extractor that was connected to the receiving bottle that had been dried to a constant weight. Two-thirds of the condensing tube volume of petroleum ether was added. The bottle was heated in a water bath so that the petroleum ether was continuously refluxed and extracted (6~8 times/h). The receiving bottle was removed after 6 h, and the petroleum ether was recovered. When 1~2 mL of the solvent remained in the receiving bottle, the bottle was dried in a water bath until the petroleum ether had evaporated. The dried bottle was placed in a drying oven at 100 ± 5 °C for 1 h and then cooled to room temperature. This procedure was repeated until the constant weight (the difference between the two weighing values) ≤2 mg. The content of crude fat was calculated using Equation (6):

Crude fat content (%) = 100 × (m_1_ − m_0_)/m_2_ (6)

where *m*_1_ is the total weight of the receiving bottle and fat after it had reached a constant weight (g), *m*_0_ is the weight of the receiving bottle (g), and *m*_2_ is the weight of the sample (g).

### 2.5. Anthocyanin Content and Composition of the Dried Samples

#### 2.5.1. Determination of the Content of Anthocyanins 

The total anthocyanin content of the sample was analyzed quantitatively as described by Dumitraşcu [19] with slight modifications. The anthocyanins have several different structures owing to their reversible transformation with the change in pH of the buffer solution, while the other interferers have no such change. The anthocyanins were present in the form of red closing cations at pH 1.0, while they were colorless semi-acetals or colorless chalcones at pH 4.5 in the buffer solution [20]. Cypermethrin-3-glucoside was usually used as the reference to quantify the anthocyanins, while most of the anthocyanins in the tested PSPs were highly glycosylated and acylated paeoniflorin anthocyanins, which played an important role in the stability of these phenolics. Therefore, paeoniflorin-3-(6′ caffeoyl-6′)-5-glucoside was selected as the reference compound in PSP in this study. According to this principle, a special pH-differential method (pH 1.0 and pH 5.5) was used to determine the content of anthocyanins in PSP. 

The extract of anthocyanins was prepared by mixing 5 g of samples with 100 mL of 60% ethanol-HCl (pH 3.0), followed by extraction in a 40 °C water bath for 120 min. After the mixture had cooled to room temperature, it was centrifuged at 4000 rpm for 20 min. The supernatant was combined and then subjected to rotary evaporation at 37 °C. The concentrated solution was filtered through a water-based microporous membrane (0.45 μm) to obtain a test solution.

A volume of 1 mL of the extracted anthocyanins sample was then diluted with 9 mL of 0.2 mol/L KCl-HCl buffer (pH 1.0) and 9 mL of 0.2 mol/L acetate-sodium buffer (pH 5.5). The diluted solution was measured using an Evolution 220 UV-VIS Spectrophotometer (Thermo Fisher Scientific, Waltham, MA, USA) at 526 nm and 700 nm, respectively. The content of anthocyanin was calculated using Equation (7):

(7) $$\mathrm{Content}\mathrm{of}\mathrm{anthocyanins}(\mathrm{mg}/100\;g)=\frac{\mathrm{Ab}}{\varepsilon }\times \mathrm{MW}\times D\times \frac{V}{G}\times 100$$

where *Ab* = (A_526nm_ − A_700nm_)_pH1.0_ − (A_526nm_ − A_700nm_)_pH5.5_, *MW* is the molecular weight of paeoniflorin-3-(6-caffeoyl-6′)-5-glucoside (1124.93 g/mol), *D* is the dilution factor, *V* is the volume of the sample (mL), *ε* is the molar absorptivity of paeoniflorin-3-(6′-caffeoyl-6′)-5-glucoside (25,311.4 mol/cm), and *G* is the quality of the sample (g).

#### 2.5.2. HPLC-DAD/ESI-MS^2^ Characterization of Anthocyanins

The difference in the profile of anthocyanins from the different varieties of PSPs was characterized by HPLC-DAD/ESI-MS^2^. The sample to be tested was passed through a 0.22 μm water-based microporous membrane before the HPLC analysis (Agilent Technologies, Santa Clara, CA, USA). The separations were conducted on a C18 column (250 mm × 4.6 mm, 5 μm). The injection volume was 10 μL, and a binary gradient elution mixture that was composed of 10% formic acid-water (A) and formic acid-acetonitrile (10/90, *v*/*v*) (B) was applied to the column as follows: 0–70 min, 20–85% B; 70–72 min, 85–100% B; 72–75 min, 100–100% B; 75–78 min, 100–20% B; and 78–80 min, 20–20% B. The flow rate for the mobile phase was 1.0 mL/min; the temperature of the column oven was set to 25 °C, and the concentration of the sample was determined at 525 nm.

The sample, which has the richest anthocyanin profile, was selected for further mass spectrometer analysis. A Thermo Fisher Accela LTQ-XL mass spectrometer equipped with an electrospray ionization ion trap mass spectrometer (ESI-MS) source was used to conduct a series of scans in the positive ion mode between *m*/*z* 100 and 2000 to obtain the data of fragment ions and mass molecular ions. The capillary voltage was set at 26 V. The drying and nebulizing gas (nitrogen) flow was 20 L/min, and its temperature was set at 270 °C.

### 2.6. Analysis of the Content of Selenium Using HG-AFS

The samples were first treated by wet digestion as described in the National Food Safety Standards in China (GB 5009.93-2017) [21]. Briefly, 0.5 g of the sample was predigested with 10 mL nitric acid–perchloric acid (9 + 1) and several glass beads for 12 h at room temperature. The solution was heated in an electric furnace until it became colorless. A volume of 5 mL of HCl (6 mol/L) was added and heated until the solution became colorless again. A volume of 2.5 mL of potassium ferricyanide (100 g/L) was added, and the mixture was followed by dilution to 10 mL with ultrapure water. 

A hydride generation-atomic fluorescence spectrometer (HG-AFS, Beijing Haiguang Instrument, Beijing, China) with a selenium hollow cathode lamp was applied to determine the content of selenium. The carrier gas was set at a flow rate of 500 mL/min, while the shielding gas was set at a flow rate of 1000 mL/min. The injection sample was 2 mL, and the time of the addition of sample, delaying, and reading was set at 8 s, 1 s, and 15 s, respectively. The temperature of atomization was set at 800 °C. The selenium content was calculated using Equation (8):

Selenium content (mg/kg) = (ρ − ρ_0_) × V/(m × 1000) (8)

where *ρ* is the mass concentration of selenium in the sample solution, *ρ*_0_ is the mass concentration of selenium in the blank solution, *V* is the volume of the sample, and *m* is the mass of the sample.

### 2.7. Statistical Analysis

Each experiment was repeated at least three times, and each test was performed three times. The data were expressed as the mean ± standard deviation (SD). SPSS 19.0 (IBM, Inc., Armonk, NY, USA) was used to analyze the data, and a one-way analysis of variance (ANOVA) was used to determine significant differences (*p* < 0.05) among the samples. The nutritional components were analyzed by principal component analysis (PCA) and cluster analysis using MetaboAnalyst 6.0.

## 3. Results and Discussion

### 3.1. Morphology and Color of the Different PSP Varieties

Color, as one of the most important senses of vision, can be used as an indicator to evaluate the quality of food. There are dominant pigments that determine the color of each food. The most important are anthocyanins (red-purple), chlorophylls (green), carotenoids (yellow-orange), and betanin (red) [22]. Table 1 illustrates the morphological and color values of transverse sections from 10 varieties of PSPs. Most varieties were oval or round, and some were spindle-shaped, including varieties ES, ZL, and XS. Moreover, they appeared to be deep purple, and there were clear white lines on the surface after the samples had been cut. This is consistent with the findings of Yong et al. [23] that the strong purple color was primarily caused by the presence of anthocyanins. The genetic difference leads to varying amounts and types of anthocyanins in different plants, which results in different shades of purple expression, while a similar color of darkness was possibly observed in the same variety. For example, the transverse sections of variety Ningzi No. 1 and its six advanced breeding lines all had a similar purple color [24]. The difference in color was further digitized by an L*a*b* color analysis. Significant differences (*p* < 0.05) for the L*a*b* values were observed among all the varieties of PSPs (Table 1). The obtained data indicated that all the varieties of PSPs had +a*/−b* values. This is consistent with the findings of Ania et al. [25], who reported that sweet potato varieties with +a*/−b* values could be described visually as purple. Additionally, the highest L* (30.9 ± 0.11), a* (28.6 ± 0.03) and b* (−2.10 ± 0.04) values were found in variety JS1. These data suggest that JS1 was more intensely red and blue than the other varieties.

### 3.2. Proximate Composition of the Different Varieties of PSPs 

The proximate composition of 10 different varieties of PSPs is shown in Table 2 in terms of moisture content (fresh samples) and crude fat, protein, starch, reducing sugar, total dietary fiber, selenium, and anthocyanin contents (dried samples). All the samples of fresh PSP had a relatively high moisture content that ranged from 60.9 to 70.1%. This finding is similar to that of Heo et al. [26]. These values suggest that tuberous plants have many intercellular spaces that can hold large amounts of cellular fluid and solutes [27]. Thus, maintaining the safe storage of fresh and active compounds will be a challenge for fresh, unprocessed PSP. 

PSPs, as a special variety of sweet potatoes, are characterized by their high carbohydrate content, low contents of fat, and protein. The content of crude fat in most varieties was between 0.5 and 0.8%, while the highest content of crude fat at 1% was identified in the variety GS. This small amount of crude fat was consistent with those of most root and tuber crops. The protein content differed significantly among the varieties (*p* < 0.05). For example, variety JS2 had a protein content (8.75 ± 0.18%) that was approximately 3-fold higher than that of variety FX (3.19 ± 0.01%). However, the levels of protein observed in this study were relatively higher than those obtained by Peksa et al. [28] when they examined 13 colored potatoes, including varieties with purple/red/yellow flesh. They reported that the protein content of potato varieties was in the range of 1~3%, and the protein quality depends on the variety, not the color of the root. 

Significant differences (*p* < 0.05) for the contents of starch, reducing sugar, and total dietary fiber were observed among all the varieties of tested PSPs (Table 2). Starch is the major carbohydrate of the PSP tuber. This is because the biosynthetic and metabolic pathways of PSPs preferentially convert carbon sources into starch and accumulate in the tuber to meet the energy needs of plants [29]. In this study, the content of starch of nine varieties of PSPs ranged from 55.2 ± 0.49% to 67.2 ± 0.81%, except for variety JS1 (43.9 ± 0.62%). These values are higher than those obtained in another study (55% on average) that involved 14 varieties of sweet potatoes, including those with purple, white, yellow, and orange flesh [30]. The value of starch content can be arranged in the following order: white sweet potatoes (61.5~67.5%) > PSP (53.0~55.9%) > yellow sweet potatoes (45.8~53.1%). 

Interestingly, it was also observed that variety ES13 had the highest starch content of 67.2 ± 0.81% and the lowest total dietary fiber content of 9.40 ± 1.42%, while variety JS1 had the lowest starch content of 43.9 ± 0.62% and the highest total dietary fiber content of 16.5 ± 1.19%. Ji et al. [31] compared the nutritional components of four different kinds of sweet potatoes, including ones that were white, red, yellow, and purple. The results showed that compared with the other types of sweet potatoes, PSPs had the highest content of total dietary fiber. All the varieties of PSPs contained some reducing sugar, which ranged from 1.44 ± 0.02% to 4.01 ± 0.02%. This is similar to the findings of Fan et al. [32], who found levels of reducing sugar as high as 5.94% and as low as 1.01% in different varieties and colors of sweet potatoes. Additionally, the soluble sugar content in the sweet potato tubers is also influenced by the specific cultivar, maturity of the tuber, and duration of storage [33].

### 3.3. Analysis of the Contents of Anthocyanins and Selenium in Different Varieties of PSPs 

There was a significant difference (*p* < 0.05) in the contents of anthocyanins and selenium among all the varieties (Table 3). The anthocyanins are considered to be the most important group of phenolics found in PSPs and contribute to their characteristic color. In this study, variety JS1 had the highest content of anthocyanins (105 ± 1.24 mg/100 g) and was approximately 2–8 folds higher than those in the other varieties. It is worth noting that JS1 showed a deeper color and had the highest values of L*a*b* values as described in Section 3.1, which also showed that the accumulation of anthocyanins affects the color of PSP flesh to some extent, thus revealing a potential link between the content of anthocyanins and visual features. In addition, some researchers also reported that PSPs had a higher content of PCP than those of the red, yellow, and white varieties of sweet potato [31], which suggests that PSPs are a good source of extracted edible anthocyanins.

Selenium (Se) plays a vital role in humans as an indispensable nutrient [34]. It was observed that variety EN possessed the highest selenium content of 15.7 ± 0.04 μg/kg. It is notable that this variety was obtained from a region with soil rich in selenium (Enshi, Hubei). The city of Enshi is known as “the world of Selenium” owing to the abundance of selenium in its soil. This characteristic soil composition significantly contributes to the accumulation of selenium in plants [35]. Therefore, this variety contains high levels of selenium, which can serve as a supplementary source of this nutrient in the daily diet. 

As two important bioactive compounds, studies of the link between selenium and anthocyanins in plant physiology represent a new research field. Research on their relationship is relatively limited, but some studies have begun to provide insight into the possible relationship between the two in plants. For example, Pu et al. [36] found a higher concentration of selenium in fruits and vegetables that are rich in pigments, which may suggest that the varieties with bright colors are more efficient at absorbing selenium. However, the direct link between selenium and anthocyanins remains to be clarified. Qing et al. [37] showed that under the influence of exogenous selenium, the concentrations of selenium, anthocyanins, chlorophyll *a*, chlorophyll *b*, and carotenoids increased during the development of colored wheat (*Triticum aestivum*) grains. After treatment with selenium, genes related to the biosynthesis of anthocyanins and other flavonoid compounds were significantly up-regulated, which promoted the accumulation of anthocyanin metabolites. This suggests that selenium may indirectly regulate the biosynthesis of anthocyanins by influencing the expression of their genes, thus, revealing the possible biological relationship between selenium and anthocyanin in plants. Although these studies provide insights into the role of selenium and anthocyanin in plants, more research is needed to further investigate the details of this correlation. 

### 3.4. Identification of the Anthocyanin Compounds of Different Varieties of PSPs

**Figure 1:**
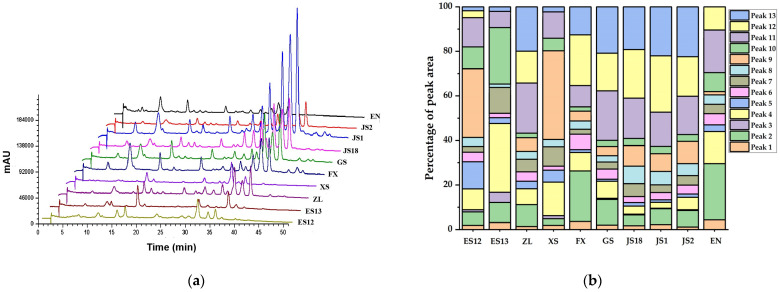
HPLC fingerprints (**a**) and percentage of 13 peak areas of anthocyanins (**b**) of 10 varieties of purple sweet potatoes.

The profile of anthocyanins in different PSP varieties was further analyzed by high-pressure liquid chromatography (HPLC). Figure 1a presents a chromatographic fingerprint of all the varieties. It was observed that all of them produced a common number of peaks (from left to right: Peak 1 to Peak 13) with different ratios. For example, varieties ES12 and XS had higher contents of peak 9, while variety ES13 had a higher content of peak 4. The proportions of peak 11, peak 12, and peak 13 were higher than those of the others in varieties ZL, FX, GS, JS1, JS2, and JS18. In contrast, the proportion of peaks 3 and 5 was relatively lower in all the samples (Figure 1b).

Moreover, variety JS1 was selected to analyze its profile of anthocyanins on account of its multitudinous peaks. The HPLC chromatogram profiles at 530 nm and their data, including the retention time, molecular ion, and main fragment ion, are shown in Table 4. A total of 13 anthocyanin monomers were isolated from JS1 using a HPLC-DAD/ESI-MS^2^ analysis. The primary compounds were peonidin-3-(-6″caffeoyl-6‴-feruloylsophoroside)-5-glucoside (Peak 13), followed by peonidin-3-caffeoylsophoroside-5-glucoside (Peak 12), peonidin-3-caffeoyl-*p*-hydroxybenzoylsophoroside-5-glucoside (Peak 11) and cyanidin-3-(6″caffeoyl-6‴-caffeoylsophoroside)-5-glucoside (Peak 9). However, there are still some differences in the composition of anthocyanins from the findings of previous studies. For example, Lee et al. [38] reported that some PSPs contained a small amount of geranium-based anthocyanins, and He et al. [39] found that some PSPs contained vanillic acid acylated cornflower and peony pigments. In this study, the primary anthocyanins observed in PSPs are composed of two types of glycosides, namely cyanidin and peonyside.

### 3.5. Correlation Analysis of Four Nutritional Components and Color in PSPs 

The contents of protein, starch, total dietary fiber, and anthocyanins were selected to evaluate their correlation with the color of PSPs. As shown in Table 5, it was noted that the anthocyanins positively correlated with the value of L*, a* and b* (*r* = 0.760, *p* < 0.05; *r* = 0.818, *p* < 0.01; *r* = 0.686, *p* < 0.05), which indicated that the content of anthocyanins had a significant influence on the color of PSPs. Wang et al. [40] reported that dark rice (*Oryza sativa* L.) had a higher content of anthocyanins than light rice, and the color value a* positively correlated with the content of anthocyanins. Thus, anthocyanins were identified as the primary substances responsible for the dark appearance of pigmented rice. In addition, the color of PSPs also positively correlated with the content of dietary fiber. Rose et al. [41] compared the percentage composition of sweet potatoes of two different colors in Rwanda. Among them, the yellow varieties had higher contents of total protein and total reducing sugars and also had higher levels of crude fiber and ash, and these varieties were nutritionally superior to the white varieties that were evaluated. 

Moreover, it was also observed that the starch content was inversely correlated with the PSP color (*r* = −0.658, *p* > 0.05). This reflects that there is an inverse relationship between the color of PSPs and their starch content. A previous study suggested that the degradation of starch may contribute to the accumulation of anthocyanins in PSPs [42]. In this line, combined with the results of Table 1, variety JS1 may have a darker purple color, better antioxidant properties, and comprehensive nutritional value because it had the lowest content of starch and the highest contents of anthocyanins and dietary fiber of all the varieties.

### 3.6. Principal Component Analysis

**Figure 2:**
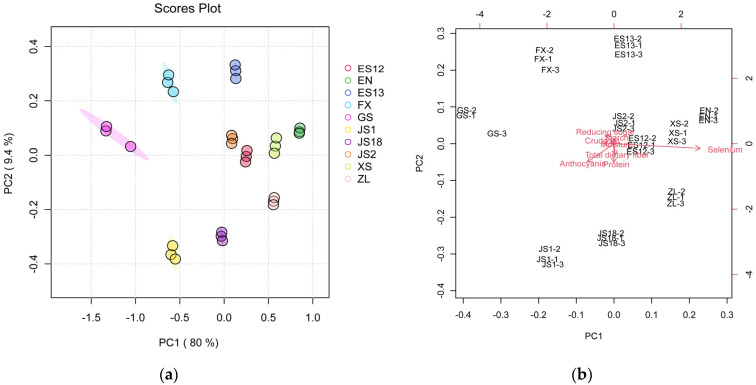
Principal component analysis (PCA) of the different varieties of purple sweet potatoes: PCA score plot (**a**); PCA biplot (**b**).

In the evaluation of the primary differences in the quality of different varieties of PSPs, a comprehensive, systematic, and scientific assessment of one or several nutritional components should be considered. PCA is an effective method for a comprehensive evaluation. In this study, eight nutrients, including the moisture, protein, crude fat, starch, reducing sugar, total dietary fiber, anthocyanins, and selenium, of 10 varieties of PSPs were analyzed using PCA, and the information provided by each PC was measured on the basis of the contributions of eigenvalues and cumulative variance. Ten varieties of PSPs were presented in the PCA score plot (Figure 2a). The first two PCs (80% for PC1 and 9.4% for PC2) explained 89.4% of the total variance. This indicates that the two PCs contain the majority of information associated with all the variables. As shown in Figure 2a, varieties EN, XS, ZL, ES12, ES13, and JS2 clustered in the positive direction of the PC1 area, while FX, JS1, JS18, and GS were located in the negative direction of this area. This phenomenon was consistent with the values of selenium and anthocyanin, which showed that all of these varieties located in the positive PC1 range had higher contents of selenium and lower ones of anthocyanin than those in the negative range of PC1 (Table 3). Moreover, these varieties of PSPs were also separated in the upper, middle, and lower areas according to the PC2, which indicated that there were still differences in the composition of the other nutrients. 

The PCA biplot (Figure 2b) was further provided to show how variables affected the PCA model. It was observed that JS, ES12, ZL, XS, and JS18 clustered in the positive ranges in PC1 and the negative range in PC2, suggesting that these varieties were more related in their protein and fiber contents. Varieties GS, FX, ES13, and EN clustered in the upper positive PC2 area, which indicated that they were more closely related in their contents of starch and reducing sugar. Variety JS1 located in the PC1 negative range and PC2 in the positive range meant that it was more closely related in its contents of anthocyanin and dietary fiber. These findings are consistent with the data shown in Table 2 and Table 3. 

### 3.7. Cluster Analysis 

**Figure 3:**
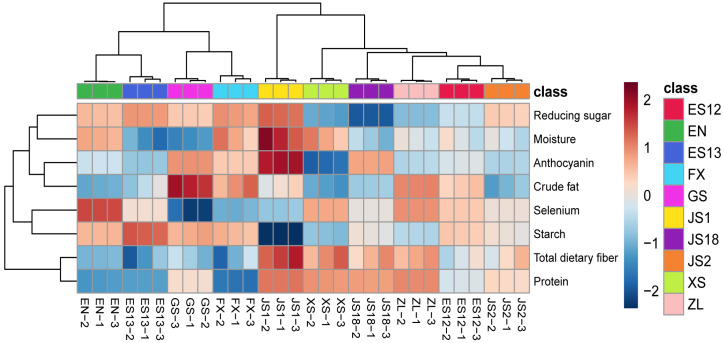
Cluster analysis of nutritional components of the different varieties of purple sweet potatoes.

Longitudinal clustering between the nutritional components and horizontal clustering between the varieties became more apparent when examined in the form of a heat map (Figure 3). The data obtained showed that the 10 varieties of PSPs that were examined could be divided into three categories. Category 1 contained five varieties of PSPs, i.e., JS2, ES12, ZL, JS18, and XS. Among them, XS, ZL, and JS18 had relatively high contents of protein and dietary fiber and a low content of starch. Consequently, they can serve as nutritional materials for the diets of diabetic patients through special food processing. Category 2 contained only one variety, JS1, which had the highest contents of anthocyanins, protein, and dietary fiber and the lowest of starch among all the varieties. It can be used as a valuable source of natural food colorants or for the development of functional foods to enhance immunity and maintain intestinal health. Category 3 contained four varieties, including FX, GS, ES13, and EN. They had higher contents of starch and reducing sugars than those of other varieties and were better for industrial use because they were suitable for producing various starch-based food products, such as noodles, cookies, and pastries. Therefore, this study provides a certain reference for the practical use and rational processing of PSP resources. 

## 4. Conclusions

In this study, the color parameters and nutritional characteristics of 10 varieties of PSPs that are cultivated in China were comprehensively evaluated to enhance their utilization and rational processing. All the varieties had a similar chemical composition, whereas the contents of nutrients differed significantly (*p* < 0.05). All the varieties of PSPs contained a mass of moisture (60.9–70.1%), starch (43.9–67.2% DW) and total dietary fiber (9.40–16.5% DW), moderate levels of reducing sugars (1.44–4.01% DW) and protein (3.19–8.75% DW), and a low content of crude fat (0.51–1.01% DW). Among them, variety JS1 had the lowest content of starch and the highest content of protein and dietary fiber. A combination of HPLC fingerprinting and HPLC-DAD/ESI-MS^2^ showed that the primary anthocyanins in PSPs are composed of cyanidin and peonyside glycosides, while the amounts and compositions were somewhat different. The higher content of anthocyanin (105 ± 1.24 mg/100 g DW) in JS1 was responsible for the significantly darker purple color. Variety EN contained the highest content of selenium (15.7 ± 0.04 μg/kg DW), which can serve as a good source for daily supplementation with selenium. Moreover, the PCA and CA suggested that varieties XS, ZL, and JS18 are desirable for developing diets for diabetic patients owing to the relatively high contents of protein and dietary fiber and the low content of starch. Variety JS1 can be used as a good resource for natural colorants or functional foods. Varieties FX, GS, ES13, and EN had higher contents of starch and reducing sugars than the other varieties, which were suitable for producing various starch-based food products such as noodles, cookies, and pastries. Taken together, this study provides some reference for the rational processing and selection of raw materials and the matching of selected varieties of PSPs. 

## Figures and Tables

**Table 1:** Morphological and color values of the transverse section in 10 varieties of purple sweet potatoes.

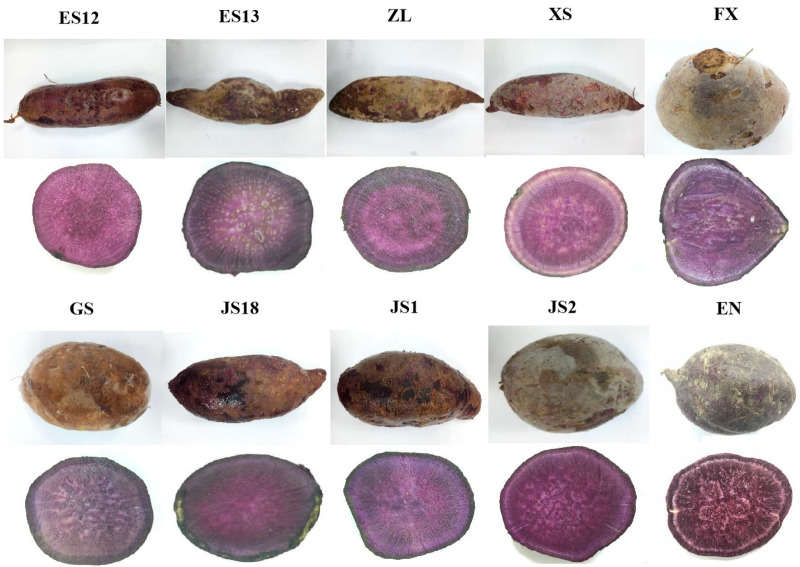				
No.	Sample ID	L*	a*	b*
1	ES12	26.6 ± 0.13 ^c^	20.8 ± 0.09 ^e^	−6.40 ± 0.03 ^h^
2	ES13	21.0 ± 0.06 ^i^	17.2 ± 0.05 ^h^	−5.20 ± 0.11 ^g^
3	ZL	23.9 ± 0.17 ^f^	18.0 ± 0.14 ^g^	−3.30 ± 0.21 ^b^
4	XS	24.4 ± 0.07 ^e^	9.30 ± 0.03 ^i^	−8.60 ± 0.07 ^i^
5	FX	22.0 ± 0.02 ^h^	26.8 ± 0.16 ^c^	−5.30 ± 0.03 ^g^
6	GS	25.9 ± 0.16 ^d^	27.8 ± 0.24 ^b^	−4.90 ± 0.18 ^f^
7	JS18	28.8 ± 0.13 ^b^	22.4 ± 0.05 ^d^	−3.60 ± 0.06 ^c^
8	JS1	30.9 ± 0.07 ^a^	28.6 ± 0.03 ^a^	−2.10 ± 0.04 ^a^
9	JS2	24.3 ± 0.11 ^e^	18.8 ± 0.14 ^f^	−4.60 ± 0.17 ^e^
10	EN	22.8 ± 0.15 ^g^	17.3 ± 0.12 ^h^	−3.90 ± 0.05 ^d^

Data in the same column with different letters were significantly different (*p* < 0.05).

**Table 2:** Nutritional components of 10 varieties of purple sweet potatoes.

No.	Sample ID	Moisture (g/100 g)	Protein (g/100 g)	Crude Fat (g/100 g)	Starch (g/100 g)	Reducing Sugar (g/100 g)	Total Dietary Fiber (g/100 g)
1	ES12	67.6 ± 0.11 ^e^	5.78 ± 0.16 ^c^	0.76 ± 0.03 ^c^	62.2 ± 0.48 ^e^	2.57 ± 0.01 ^d^	12.1 ± 1.06 ^ab^
2	ES13	64.1 ± 0.38 ^c^	3.94 ± 0.09 ^b^	0.61 ± 0.07 ^ab^	67.2 ± 0.81 ^f^	3.77 ± 0.05 ^h^	9.40 ± 1.42 ^a^
3	ZL	66.3 ± 0.14 ^d^	8.72 ± 0.04 ^e^	0.87 ± 0.01 ^c^	55.6 ± 0.25 ^bc^	2.14 ± 0.02 ^c^	14.5 ± 0.72 ^bcd^
4	XS	70.1 ± 0.19 ^g^	8.63 ± 0.01 ^e^	0.51 ± 0.01 ^a^	55.2 ± 0.49 ^b^	2.02 ± 0.02 ^b^	15.7 ± 1.50 ^cd^
5	FX	66.3 ± 0.50 ^d^	3.19 ± 0.01 ^a^	0.83 ± 0.09 ^c^	60.3 ± 0.65 ^d^	3.62 ± 0.03 ^g^	9.70 ± 1.82 ^a^
6	GS	60.9 ± 0.32 ^a^	6.16 ± 0.13 ^c^	1.01 ± 0.05 ^d^	60.1 ± 0.01 ^d^	3.10 ± 0.05 ^e^	10.3 ± 0.83 ^a^
7	JS18	62.0 ± 0.18 ^b^	7.81 ± 0.09 ^d^	0.54 ± 0.01 ^a^	55.7 ± 0.17 ^bc^	1.44 ± 0.02 ^a^	13.5 ± 1.30 ^bc^
8	JS1	67.0 ± 0.16 ^de^	8.75 ± 0.18 ^e^	0.66 ± 0.05 ^b^	43.9 ± 0.62 ^a^	4.01 ± 0.02 ^i^	16.5 ± 1.19 ^d^
9	JS2	63.7 ± 0.47 ^c^	6.41 ± 0.04 ^c^	0.51 ± 0.04 ^a^	56.7 ± 0.35 ^c^	3.09 ± 0.01 ^e^	12.6 ± 1.30 ^ab^
10	EN	68.8 ± 0.12 ^f^	3.87 ± 0.08 ^b^	0.52 ± 0.01 ^a^	62.8 ± 0.37 ^e^	3.41 ± 0.03 ^f^	10.2 ± 0.12 ^a^

Values are expressed as mean ± standard deviation (*n* = 3); mean values that are not significantly different from each other (*p* > 0.05) are represented by the same letter. Here, the moisture content is calculated on a wet basis, while the content of other substances is calculated on a dry basis.

**Table 3:** The contents of anthocyanin and selenium in 10 varieties of purple sweet potatoes.

NO.	Sample ID	Anthocyanin (mg/100 g)	Selenium (mg/kg)
1	ES12	33.8 ± 1.06 ^f^	3.90 ± 0.03 ^g^
2	ES13	22.9 ± 0.64 ^b^	2.57 ± 0.08 ^f^
3	ZL	27.2 ± 0.85 ^d^	7.63 ± 0.09 ^i^
4	XS	13.2 ± 0.73 ^a^	5.99 ± 0.03 ^h^
5	FX	44.9 ± 0.79 ^g^	0.53 ± 0.05 ^b^
6	GS	59.7 ± 0.56 ^i^	0.11 ± 0.04 ^a^
7	JS18	54.8 ± 1.16 ^h^	2.03 ± 0.03 ^d^
8	JS1	105 ± 1.24 ^j^	0.73 ± 0.07 ^c^
9	JS2	25.3 ± 0.57 ^c^	2.26 ± 0.06 ^e^
10	EN	30.8 ± 0.34 ^e^	15.7 ± 0.04 ^j^

Values are expressed as mean ± standard deviation (*n* = 3); mean values that are not significantly different from each other (*p* > 0.05) are represented by the same letter.

**Table 4:** Chromatogram and mass data of the anthocyanins in variety JS1.

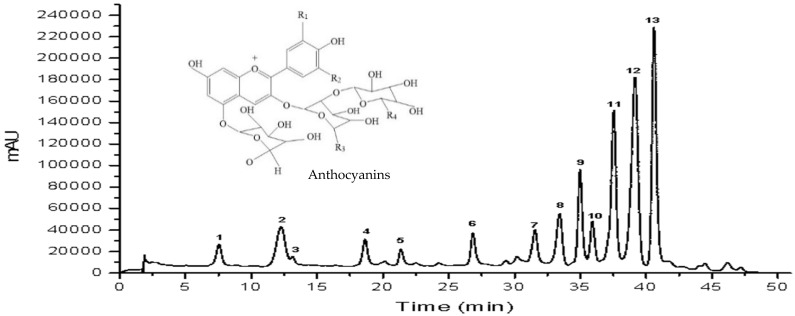				
Peak No.	*t_R_* (min)	[M]^+^ (*m/z*)	Fragment (*m/z*)	Compounds
1	7.56	773	611, 449, 287	Cyanidin-3-sophoroside-5-glucoside
2	12.27	787	625, 463, 301	Peonidin-3-sophoroside-5-glucoside
3	13.25	893	731, 449, 287	Cyanidin-3-p-hydroxybenzoylsophoroside-5-glucoside
4	18.66	907	745, 463, 301	Peonidin-3-p-hydroxybenzoylsophoroside-5-glucoside
5	21.38	949	787, 449, 287	Cyanidin-3-(6″-feruloylsophoroside)-5-glucoside
6	26.85	963	949, 463, 301	Peonidin-3-feruloylsophoroside-5-glucoside
7	31.56	1055	893, 449, 287	Cyanidin-3-(6″-caffeoyl-6‴-p-hydroxybenzoylsophoroside)-5-glucoside
8	33.43	935	773, 449, 287	Cyanidin-3-caffeoylsophoroside-5-glucoside
9	34.98	1111	949, 463, 301	Cyanidin-3-(6″-caffeoyl-6‴-caffeoylsophoroside)-5-glucoside
10	35.92	1101	939, 463, 301	Peonidin-3-dicaffeoylsophoroside-5-glucoside
11	37.54	1069	907, 463, 301	Peonidin-3-caffeoyl-p-hydroxybenzoylsophoroside-5-glucoside
12	39.16	949	787, 463, 301	Peonidin-3-caffeoylsophoroside-5-glucoside
13	40.60	1125	963, 463, 301	Peonidin-3-(6″-caffeoyl-6‴-feruloylsophoroside)-5-glucoside

**Table 5:** Correlation analysis of the nutritional components and color of purple sweet potatoes.

	L*	a*	b*	Protein	Starch	Total Dietary Fiber	Anthocyanin
L*							
a*	0.415						
b*	0.453	0.646					
Protein	0.640	−0.237	0.219				
Starch	−0.749	−0.236	−0.479	−0.757 *			
Total dietary fiber	0.635	−0.247	0.166	0.923 **	−0.852 **		
Anthocyanin	0.760 *	0.818 **	0.686 *	0.227	−0.658	0.270	

* indicates a significant correlation at the level of 0.05; ** indicates a significant correlation at the level of 0.01.

## Data Availability

The original contributions presented in the study are included in the article, further inquiries can be directed to the corresponding authors.
